# Profiles of Metabolic Genes in *Uncaria rhynchophylla* and Characterization of the Critical Enzyme Involved in the Biosynthesis of Bioactive Compounds-(iso)Rhynchophylline

**DOI:** 10.3390/biom12121790

**Published:** 2022-11-30

**Authors:** Mengquan Yang, Bowen Yao, Rongmei Lin

**Affiliations:** 1 College of Tobacco Science, Henan Agricultural University, Zhengzhou 450002, China; 2 School of Science, Beijing University of Chemical Technology, Chaoyang District, Beijing 100029, China; 3 Graduate School of Pharmaceutical Sciences, The University of Tokyo, Bunkyo-ku, Tokyo 113-0033, Japan; 4 College of Plant Protection, Henan Agricultural University, Zhengzhou 450002, China

**Keywords:** *Uncaria rhynchophylla*, spiroindole alkaloids, biosynthetic pathway, transcriptome, rhynchophylline and isorhynchophylline

## Abstract

Rhynchophylline (RIN) and isorhynchophylline (IRN), two of the representative types of indole alkaloids, showed the unique spiroindole structures produced in *Uncaria rhynchophylla*. As the bioactive constituent of *U. rhynchophylla*, IRN has recently drawn extensive attention toward antihypertensive and neuroprotective activities. Despite their medicinal importance and unique chemical structure, the biosynthetic pathways of plant spiroindole alkaloids are still largely unknown. In this study, we used *U. rhynchophylla*, extensively used in traditional Chinese medicine (TCM), a widely cultivated plant of the *Uncaria* genus, to investigate the biosynthetic genes and characterize the functional enzymes in the spiroindole alkaloids. We aim to use the transcriptome platform to analyse the tissue-specific gene expression in spiroindole alkaloids-producing tissues, including root, bud, stem bark and leaf. The critical genes involved in the biosynthesis of precursors and scaffold formation of spiroindole alkaloids were discovered and characterized. The datasets from this work provide an essential resource for further investigating metabolic pathways in *U. rhynchophylla* and facilitate novel functional enzyme characterization and a good biopharming approach to spiroindole alkaloids.

## 1. Introduction

*Uncaria rhynchophylla* (*U. rhynchophylla*, Gouteng in Chinese) is a model member of the *Uncaria* genus, which belongs to the *Rubiaceae* family [1]. The major indole alkaloids rhynchophylline (RIN) and isorhynchophylline (IRN) were isolated from *U. rhynchophylla* and other Uncaria plants and investigated a lot by natural product researchers [1]. *U. rhynchophylla*, a traditional Chinese herbal drug, has been used for hypertension treatment [1,2]. It was proven that crude extracts containing indole alkaloids could improve the cognitive function of mice. It has potential as an Alzheimer’s disease treatment [3]. As the critical medicinal composition, RIN and IRN can be used for neuronal differentiation [4] and exhibit similar antihypertensive effects with different targets [5]. Especially, IRN showed neuroprotective ability by inhibiting intracellular calcium overload and tau protein hyperphosphorylation [6,7].

Due to its medicinal importance, *U. rhynchophylla* is widely planted in south China. As the representative ingredients, RIN (28–50%) and IRN (15%) accounted for a large part of the total alkaloids [8]. However, the biosynthetic pathways of RIN and IRN are still not elucidated biochemically. Monoterpene indole alkaloids, as a large class of plant natural products, have more than 2000 members were isolated and characterized in various plants, such as *Apocynaceae* family and *Rubiaceae* family plants [9]. Most plant indole alkaloids, such as vinblastine and vincristine, originate from the common biosynthetic intermediate strictosidine through the condensation of secologanin and tryptamine. In *Catharanthus roseus*, one of the indole alkaloids-producing plants, strictosidine biosynthesis has been well studied [10]. However, the biosynthesis of RIN and IRN in *U. rhynchophylla* is still not studied, and no enzyme was characterized in the biosynthesis. Here, only crude protein results proposed that *Ur*STR can accept N-w-methyltryptamine as the substrate for N-methylstrictosidine formation [11].

To understand the formation of major bioactive ingredients in the *Uncaria* capsule extracts, *Uncaria* capsules RNA samples were used for transcriptome sequencing in 2014 [12]. Recently, the transcriptomic data for leaves were reported to investigate the regulatory mechanism of ethylene [13]. However, only the nucleotides from the chloroplast genome of *U. rhynchophylla* are available in the NCBI database. Up to now, the genomic data is highly limited, which heavily hampers the biosynthetic study of spiro-indole alkaloids in *U. rhynchophylla*, and the biosynthetic pathway of RIN and IRN remains to be elucidated.

**Figure 1:**
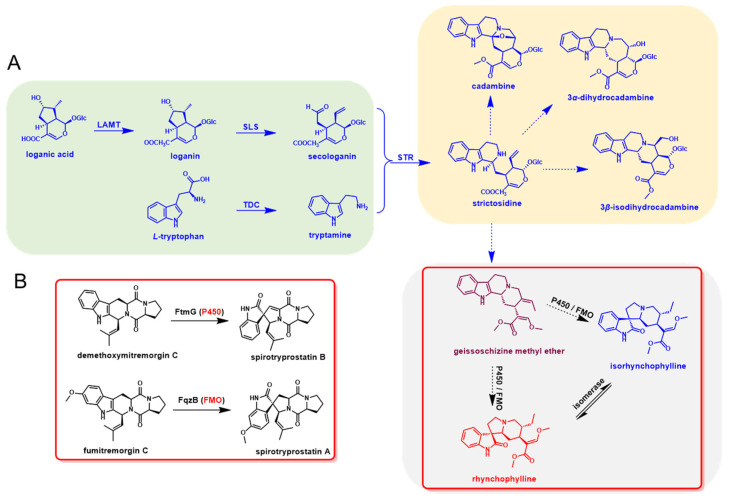
Proposed biosynthetic pathway of rhynchophylline and isorhynchophylline. (**A**) Metabolites and enzymes involved in the indole alkaloids biosynthetic pathway in *U. rhynchophylla*. (**B**) The homologs (P450 and FMO) for spiro-indole alkaloids formation in microbial natural product biosynthesis.

The early step for the biosynthesis of indole alkaloids in *U. rhynchophylla* should be similar to other indole alkaloids, which contain the tryptamine and iridoid pathways. After the condensation of tryptamine and secologanin, the spiroindole alkaloids were derived by further oxidation and modification of strictosidine [12]. In the late step, cytochrome P450s (P450), flavin-containing monooxygenases (FMO) and isomerases were most likely involved in the RIN and IRN formation [14,15]. The whole biosynthetic pathway was proposed and divided into the early and late steps (Figure 1).

RNA-seq is the most efficient approach for non-model plants, especially medicinal plants, which can quickly provide genomic information and facilitate functional gene screening [16,17]. After identifying the natural products in *U. rhynchophylla*, we constructed a transcriptomic dataset to investigate the gene function and gene expression profiles in *U. rhynchophylla*. We evaluated the gene expression in different tissues (root, leaf, young bud and stem bark) and proposed the biosynthetic pathways of RIN and IRN in *U. rhynchophylla*. Furthermore, *Ur*STR was characterized by the biochemical approach; N-methyltryptamines were excluded as possible *Ur*STR substrates. This work will be a valuable genetic resource for biopharming and the elucidation of the biosynthetic pathway of spiroindole alkaloids. It sheds light on the synthetic biology research on spiroindole alkaloids.

## 2. Results

### 2.1. Specific Indole Alkaloids in U. rhynchophylla

**Figure 2:**
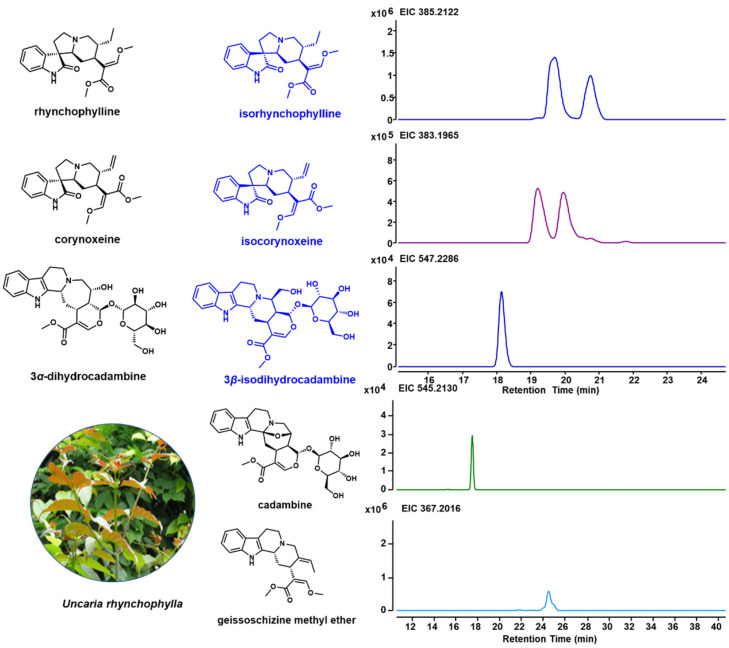
Specific indole alkaloids in *U. rhynchophylla* and LC-MS spectrum for indole alkaloids. Structures for rhynchophylline, corynoxeine and 3*α*-dihydrocadambine are shown in black, and their isomers (isorhynchophylline, isocorynoxeine, and 3*β*-dihydrocadambine) are shown in blue.

Due to the unique indole alkaloids in *U. rhynchophylla*, non-target metabolite profiling was performed using LCMS. Based on the previous studies [1,18,19], spiroindole alkaloids (rhynchophylline, isorhynchophylline, corynoxeine and isocorynoxeine) and several indole alkaloids (3*α*-dihydrocadambine, 3*β*-dihydrocadambine, cadambine and geissoschizine methyl ether) in shunt pathways were detected (Figure 2). In addition, the TIC profiles for different tissues are shown in Supplementary Figure S2, and all indole alkaloids annotated by Compound Discovery are shown in Supplementary Table S3.

### 2.2. De Novo Transcriptome of U. rhynchophylla

**Figure 3:**
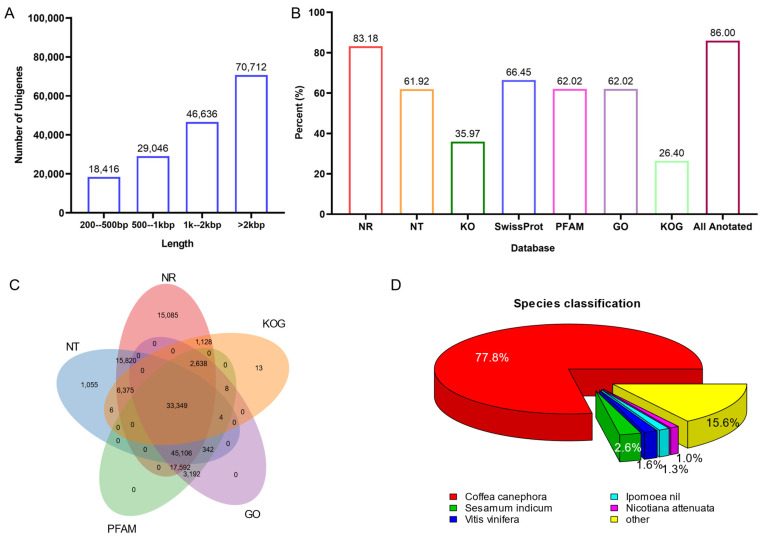
(**A**) the distribution of unigene length. (**B**) unigenes annotation by public databases. (**C**) Veen diagram of unigene annotation. (**D**) distribution of annotated species.

To further understand the genetic basis of indole alkaloid biosynthesis in *U. rhynchophylla*, four tissues from plants were collected for RNA-seq. Deep RNA sequencing and transcriptome analysis were performed. A total of 241.5 million raw reads were generated, and 35.44 Gb of clean bases were obtained for the transcriptome assembly. Among them, 10.45 Gb from leaves, 8.32 Gb from stem bark, 8.21 Gb from roots, and 8.46 Gb from buds were obtained, respectively (Table 1). After transcriptome assembly, 311,204 unigenes (longer than 500 bp) were generated, and the N50 value of all unigenes’ length is 2887 bp (Table 1). The distribution of unigene length is shown in Figure 3A, and 70,712 unigenes are longer than 2000 bp.

### 2.3. Gene Expression Comparison among Tissues

The gene expression levels of all unigenes in four tissues are calculated and evaluated by fragment per kilobase per million mapped reads (FPKM) in the four tissues. It showed similar FPKM values among all the tissues (Supplementary Figure S1).

### 2.4. Functional Annotation for U. rhynchophylla

All unigenes were searched against the Nr, Nt and SWISS-PROT databases using BLASTX. Unigenes were also compared with the Protein Families database (PFAM), the Clusters of Orthologous Genes (COG) and the Kyoto Encyclopedia of Genes and Genomes (KEGG) using BLASTX. InterProScan5 annotated InterPro domains and functional assignments were mapped onto the Gene Ontology (GO) database. Figure 3B and Table 2 show that 86.0% (141,747) of unigenes were annotated in at least one database. As shown in Figure 3C, 33,349 unigenes are commonly annotated in five public databases. After aligning with all the genes in the databases, *Coffea canephora* (77.8%) showed the highest number of best hits (Figure 3D). It is pretty consistent with phylogenesis. Since both *C. canephora* and *U. rhynchophylla* belong to the *Rubiaceae* family, and *C. canephora* is the closest to *U. rhynchophylla* with sequenced genomic resources.

### 2.5. GO and KEGG Classification

**Figure 4:**
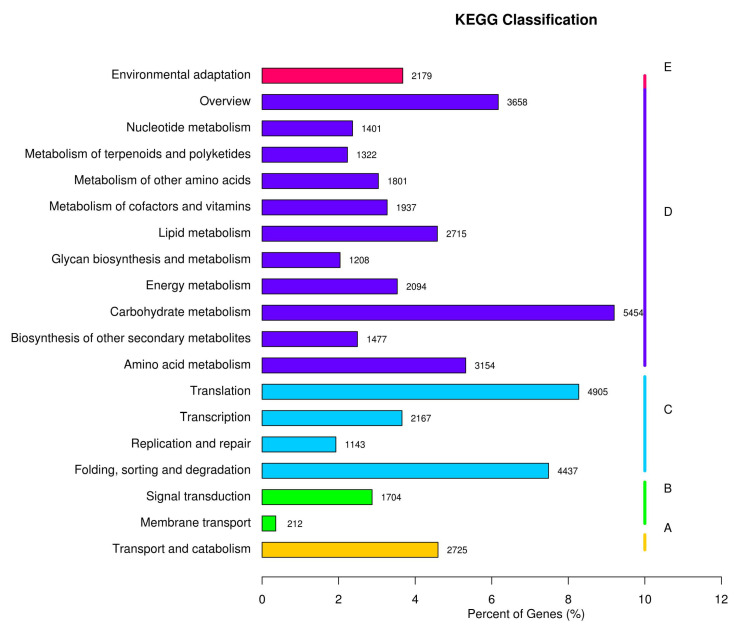
KEGG pathway annotation. (**A**) cellular processes; (**B**) environmental information processing; (**C**) genetic information processing; (**D**) metabolism; and (**E**) organismal systems.

Based on the KEGG annotation, all the unigenes are classified into five main categories (Figure 4, A, Organismal Systems; B, Metabolism; C, Genetic Information Processing; D, Environmental Information Processing; E, Cellular Processes). Among them, 4955 unigenes were annotated with the metabolism of amino acids, and 1477 unigenes were annotated with the biosynthesis of secondary metabolites, related to the natural product biosynthesis.

**Figure 5:**
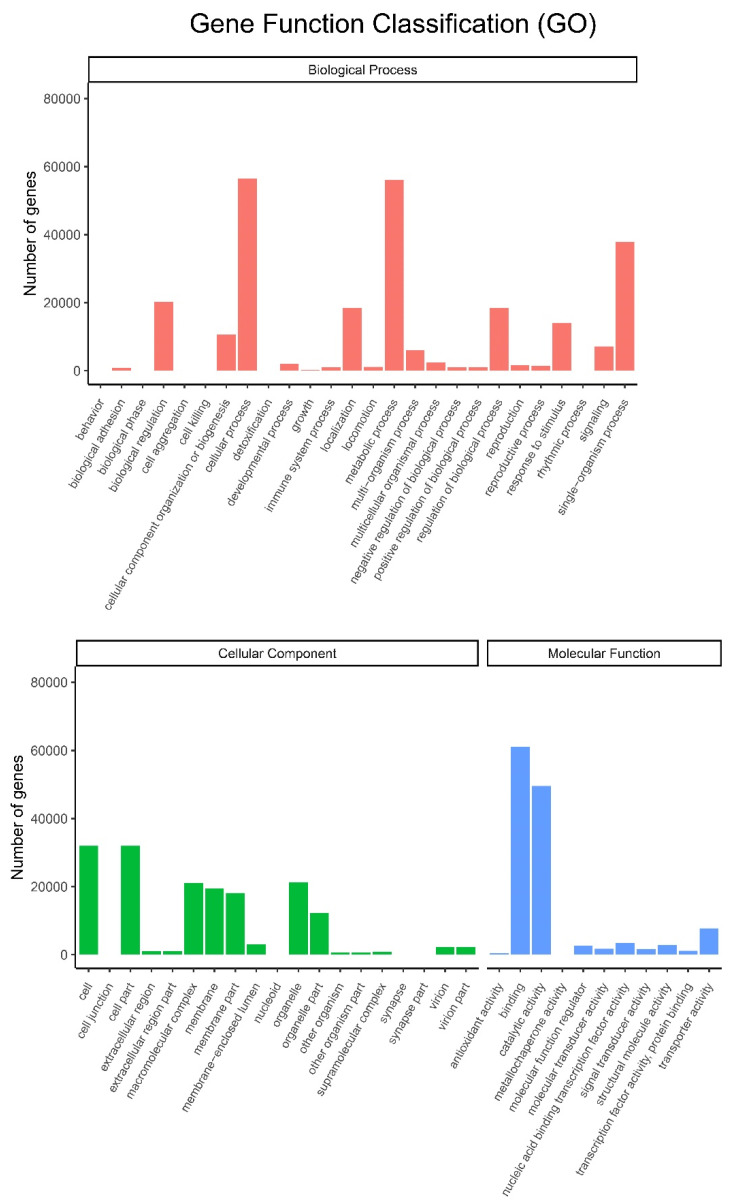
Histogram of GO classifications. Upper: biological process; Lower: cellular component and molecular function.

All the unigenes were assigned to different categories, including biological process, cellular component and molecular function (Figure 5). After annotation by the GO database, all these three major categories were classified into 56 subgroups. Cellular process and metabolic process subgroups in biological process, cell and cell part subgroups in cellular component, and binding and catalytic activity subgroups in molecular function were dominant in each category. Among them, metabolic processes and catalytic activity subgroups are involved in the biosynthesis of small molecules.

### 2.6. Transcription Factors

**Figure 6:**
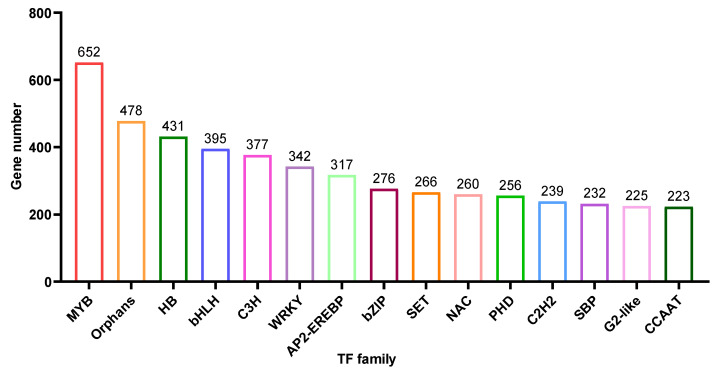
Transcription Factor (TF) were predicted into 80 subgroups, and the histogram showed the abundance of the top 15 subgroups.

Based on the unigene annotation and prediction, 8,891 unigenes were predicted to be transcriptional factors (TF) (Table S1). Here, we showed the top 15 TF types (Figure 6), MYB (652 members), Orphans (478 members), HB (431 members), bHLH (395 members), C3H (377 members), WRKY (342 members) and AP2-EREBP (317 members) are the most abundant and contain more than 300 members.

### 2.7. Phylogenetic Analysis

**Figure 7:**
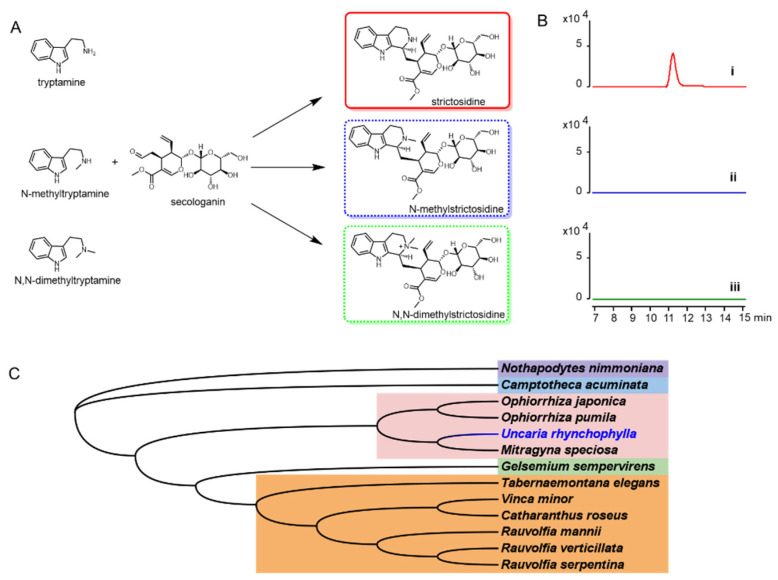
(**A**) tryptamine and analogues were proposed to be accepted by *Ur*STR for strictosidine and analogue formation. (**B**) biochemical characterization of *Ur*STR in vitro. (i) with tryptamine, (ii) with N-methyltryptamine and (iii) with N-dimethyltryptamine. (**C**) phylogenetic tree of STRs from indole alkaloids producing plants.

To characterize the *Ur*STR, the *Cr*STR was used as the probe to blast the transcriptome database. The gene encoding *Ur*STR was cloned and used for in vitro assays (Figure 7A,B). All the reported STRs from other indole alkaloids-producing plants were used for phylogenetic relationship analysis. *Ur*STR was closely related to STRs from *Ophiorrhiza pumila*, *Ophiorrhiza japonica* and *Mitragyna speciosa* (Figure 7C).

### 2.8. STR Activity

Dolichantoside, N-methylstrictosidine, is reported as the primary indole alkaloid in the stem bark of *Strychnos tricalysioides* [20]. Dolichantoside was also found in *Uncaria tomentosa* and can be stimulated after H_2_O_2_ elicitation. Moreover, the crude proteins from root culture showed the ability to convert N-methyltryptamine and secologanin into N-methylstrictosidine [11]. The authors proposed that the strictosidine synthases from *U. tomentosa* might catalyse this reaction. Up to now, strictosidine synthases that can accept N-methyltryptamine as a substrate have not reported. The strictosidine synthase in *U. tomentosa* might be an exception. Based on these results, we isolated the cDNA from *U. rhynchophylla*, expressed the gene and purified the protein. To test the activity of *Ur*STR, tryptamine, N-methyltryptamine and N, N-dimethyltryptamine were used as the substrates. After being supplied with secologanin, only strictosidine was detected by LCMS (Figure 7A,B).

### 2.9. The Acceptance of Tryptamine and Analogues

**Figure 8:**
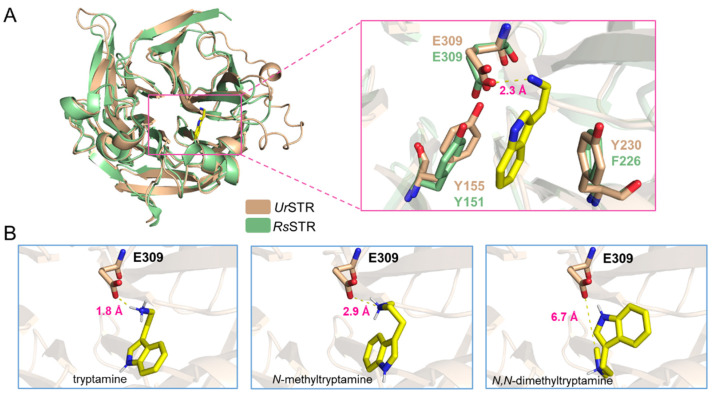
Docking results for tryptamine and analogues. (**A**) the structure of *Ur*STR in wheat and *Rs*STR in pale green and tryptamine in the active pocket. (**B**) the distance between the substrate (tryptamine, N-methyltryptamine and N,N-dimethyltryptamine) and the catalytic residue Glu309 (E309).

As shown in Figure 7, N-methyltryptamines cannot be accepted by *Ur*STR, which is not consistent with the previous crude protein assay in *U. tomentosa*. Due to the close relationship between *Ur*STR and *Rs*STR, we docked the substrates (tryptamine and analogues) into the *Ur*STR structure, which AlphaFold2 predicted. After comparing with *Rs*STR, the docking study explained the unacceptance of N-methyltrypatmines well (Figure 8). Indole surfaces of tryptamine are located in parallel with the benzene rings of Tyr155 (Y155) and Tyr230 (Y230) to form sandwich-like structure arrangements, which is similar to *Rs*STR [21]. Glu309 (E309), as the catalytic residue, interacts with the nitrogen atom on the side chain of tryptamine and its analogues. We calculated the distance between the substrates (tryptamine, N-methyltryptamine and N-dimethyltryptamine) and Glu309 (E309). The results showed that the nitrogen atom on N-methyltryptamine and N-dimethyltryptamine is far from E309, which might result in the inability to accept N-methyltryptamine and N,N-dimethyltryptamine.

### 2.10. Candidate Genes in Spiroindole Alkaloids Formation

**Figure 9:**
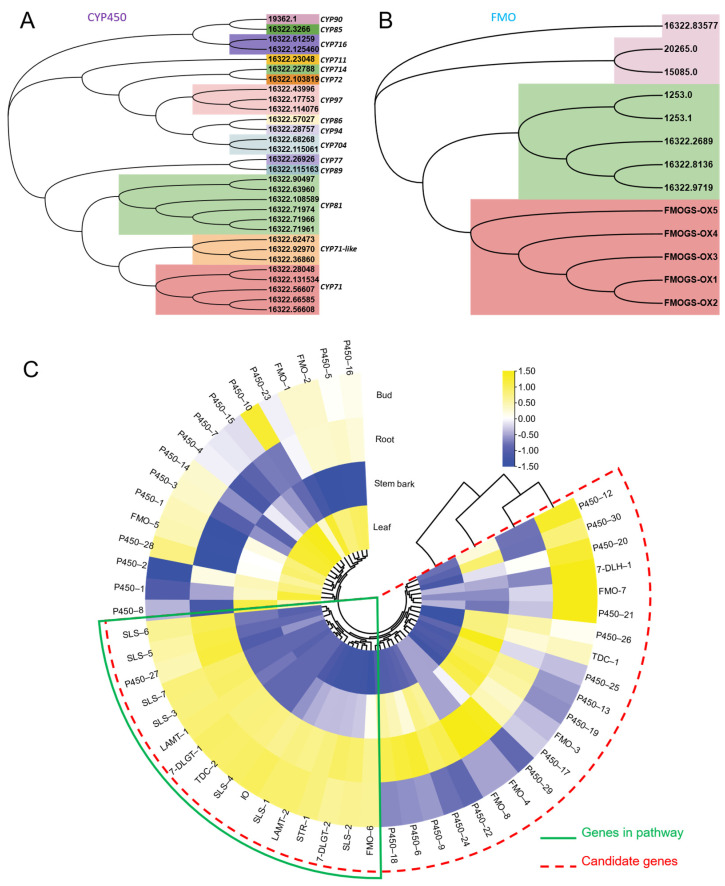
Candidate CYP450s and FMOs involved in spiroindole alkaloids. Phylogenetic analysis of CYP450s (**A**) and FMOs (**B**). (**C**) the co-expression analysis of candidate CYP450s and FMOs.

As shown in Figure 1B, FtmG (a P450 enzyme) and FqzB (a FMO enzyme) are reported to be involved in microbial spiroindole alkaloid (spirotryprostatin A and B) formation [14]. These enzymes involved in the biosynthesis of spiroindole alkaloids give us clues to investigate the enzymes involved in plant spiroindole alkaloids. Based on the gene function and annotation, those P450s and FMOs were selected as the candidates for plant spiroindole alkaloids biosynthesis and used for phylogenetic analysis (Figure 9A,B). Thirty CYP450s were assigned to 15 CYP450 families, CYP71, CYP71-like and CYP81 enzymes are dominant (Figure 9A). Seven FMOs were identified by using FMO (FMOGS-OX1-5) in *Arabidopsis thaliana* (Figure 9B). All these CYP450s and FMOs were used for co-expression analysis with the homologs of the reported genes (7-deoxyloganetic acid UDP-glucosyltransferase (7-DLGT), 7-deoxyloganic acid hydroxylase (7-DLH, catalysed by CYP72A224), secologanin synthase (SLS), tryptophan synthase β (TSB), tryptophan decarboxylase (TDC) and strictosidine synthase (STR)) in *U. rhynchophylla*. The genes in the early steps of indole alkaloids are abundant in bud and root. It indicates that those CYP450s and FMOs are possibly involved in spiroindole alkaloids, which are highly expressed in the bud or root (Figure 9C).

## 3. Discussion

### 3.1. U. rhynchophylla Produces Specific Spiroindole Alkaloids

According to previous studies, *Uncaria* is a good producer of indole alkaloids, which are widely used for disease treatment [18]. Among them, *U. rhynchophylla* is well studied and widely cultivated as a herb in China that is used for traditional medicine production and active ingredient isolation. *U. rhynchophylla* (3 years old) used in this study was acquired from the market in China and cultivated in a greenhouse (under 16 h of light and 8 h of dark at 25 °C), which was identified by Prof. Yuehong Yan. A voucher specimen (UR201905) has been deposited in the laboratory of Chemical Biology and Biochemistry, the Shanghai Institute of Plant Physiology and Ecology. Here, we performed the non-target metabolites profiling of *U. rhynchophylla*, spiroindole alkaloids (rhynochophylline, isorhynochophylline, corynoxeine and corynoxeine) and some indole alkaloids in the shunt pathway (3α-dihydrocadambine, 3β-dihydrocadambine, cadambine and geissoschizine methyl ether) were detected. All of these indole alkaloids represent the specific indole alkaloids in *Uncaria* plants, which proved that *U. rhynchophylla* is a good model for the biosynthetic study of spiroindole alkaloids and other *Uncaria*-specific indole alkaloids. The alkaloids mainly act on the cardiovascular system and central nervous neurological systems, which possess hypotensive, antiarrhythmic and sedative properties. Among them, 3α-dihydrocadambine and geissoschizine methyl ether exhibited dose-dependent hypotensive and anti-hypertensive effects. The spiroindole alkaloids rhynchophylline, isorhynchophylline, corynoxeine and isoorynoxeine showed neuroprotective effects [19].

### 3.2. Candidate Genes Involve or Regulate Spiroindole Alkaloids Biosynthesis

Based on KEGG classification (Figure 4), unigenes in the biosynthesis of other secondary metabolites and amino acid mechanism terms are always related to natural product biosynthesis. The pathways of indole alkaloids biosynthesis (in the biosynthesis of other secondary metabolites term) and tryptophan metabolism and biosynthesis (in amino acid metabolism term) are pretty closely related to indole alkaloids formation [22,23]. As shown in Figure 6, lots of TFs were identified in *U. rhynchophylla*, that are reported to regulate the production of indole alkaloids. MYB [24], WRKY [25], AP2/ERF [26] in *Ophiorrhiza pumila* and bHLH [27] in *Catharanthus roseus* are already characterized as the regulators in camptothecin, catharanthine, tabersonine and ajmalicine production. It indicates that TFs in MYB, WRKY, AP2/ERF and bHLH are likely to regulate spiroindole alkaloid production in *U. rhynchophylla*. In addition, the members of the CYP71 and CYP81 families are usually involved in indole alkaloid, flavonoid, and terpenoid metabolism [28]. The unigenes of the CYP71 and CYP81 families shown in Figure 9 are also possible candidates for spiroindole alkaloid biosynthesis.

### 3.3. N-Methyltryptamines Are Not the Natural Substrates for N-Methylstrictosidine Formation

*Uncaria tomentosa* is one species of *Uncaria* plants, and the authors proved that the crude protein from the root could convert N-methyltryptamine and secologanin into N-methylstrictosidine [11]. These results attracted our attention, which is the only evidence for N-methylstrictosidine by strictosidine synthases. *U. rhynchophylla* is closely related to *U. tomentosa*; we cloned the responsible gene and purified the enzyme to characterize the function of *Ur*STR. It showed that *Ur*STR could accept only tryptamine, and N-methyltryptamines (N-methyltryptamine and N,N-dimethyltryptamine) could not be accepted (Figure 7A,B). After docking analysis based on *Rs*STR [21], the critical residue Glu309 (E309) is far away from the nitrogen atom on the amino group of N-methyltryptamines, which can explain this phenomenon well (Figure 8). Here, we exclude the possibility of *Ur*STR accepting N-methyltryptamines.

### 3.4. Biosynthesis of Spiroindole Alkaloids in U. rhynchophylla

It was reported that FtmG (CYP450) and FqzB (FMO) are involved in microbial spiroindole alkaloid biosynthesis [14]. Based on this clue, we investigated the enzymes involved in plant spiroindole alkaloids. After phylogenetic and co-expression analysis, we selected a series of candidate CYP450s and FMOs involved in spiroindole alkaloids *U. rhynchophylla* (Figure 9). The combinational analysis of phylogenetic analysis (Figure 9A,B) and correlation analysis (Figure 9C) with genes in the early step of indole alkaloids helps us narrow down the candidates; it is an excellent way to characterize these enzymes by knocking them out or in vitro assaying. In addition, when the contents of RIN and IRN from different tissues were compared, it showed highest content in stem bark among all the tissues, followed by the bud, leaf and root (Supplementary Figure S3). Based on the correlation analysis (Figure 9C), since the genes involved in the early step of indole alkaloids were highly expressed in leaves and roots, we proposed two possibilities: (1) genes involved in the late steps of spiroindole alkaloids are also mainly synthesised at the same tissues (leaf and root) and then transferred to stem bark by transporters; (2) genes involved in the late steps might be highly expressed in stem bark, and the spiroindole alkaloids were mainly synthesised in stem bark.

### 3.5. Global Transcriptome Provides a Valuable Genetic Resource for the Biosynthetic Pathway Elucidation of Indole Alkaloids

Several biosynthetic pathways of plant indole alkaloids were recently elucidated, such as cathranthine, ibogaine and strychnine [29,30]. In biosynthetic pathway elucidation, transcriptome sequencing and differential expression analysis provide the gene candidates for subsequent studies [22,29,31]. Here, we reported the global transcriptome of *U. rhynchophylla,* which is strongly related to spiroindole alkaloids biosynthesis [18]. As noted, the famous anti-cancer drug vinblastine was synthesised in yeast by a synthetic biology approach after all the enzymes involved in the biosynthetic pathway were elucidated [23]. Our results provide a valuable resource for the biosynthetic study and also pave the way for spiroindole alkaloids’ production by the synthetic pathway approach.

## 4. Materials and Methods

### 4.1. Plant Materials

*U. rhynchophylla* plants were cultivated in the greenhouse (under 16 h of light and 8 h of dark at 25 °C). The root, bud, stem bark and leaves from five different plants at similar growth stages were separated and collected in clean tubes. These samples were immersed in liquid nitrogen and stored at −80 °C for metabolite analysis and RNA extraction.

### 4.2. Non-Targeted Metabolites Analysis

The samples (including the root, bud, stem bark and leaves) were ground into powder under liquid nitrogen for metabolite extraction and LC-MS analysis. Then, 1 mL methanol was added into the tubes containing ~50 mg powder, and the mixtures were vortexed for 5 min, followed by extraction by ultrasonication at 4 °C for 1 h in an ice bath for 1 h. All samples were centrifuged at 4 °C at 20,000× *g* for 30 min, filtered through a 0.22-μm filter membrane and injected 2 μL of each sample into an Agilent 1290 UHPLC system coupled with an Agilent 6545 Q-TOF ESI high-resolution mass spectrometer (HRMS) for analysis. The column used for separation was an Agilent 300 Extend-C18 (4.6 × 150 mm, 3.5 μm), with the temperature set to 40 °C. Mobile phases A (H_2_O + 0.1% formic acid) and B (acetonitrile + 0.1% formic acid). The mass spectrometer parameters were set as the same as in the previous study [32].

### 4.3. Transcriptome Sequencing

Total RNA was extracted from four tissues (root, bud, stem bark and leaves). The total RNA samples for the specific tissue from five individual plants were mixed, respectively. The TIANGEN RNAprep Pure Plant Kit was used for the RNA extraction according to the manufacturer’s instructions. DNase I was used for the contaminated DNA digestion. The purified total RNA was quantified using a NanoDrop, and agarose gel electrophoresis. Oligo(dT) was used to isolate mRNA, and then the mRNA was fragmented by mixing it with the fragmentation buffer. cDNA was synthesised using the mRNA fragments as templates. After PCR amplification of cDNA and quantification, the cDNA library was sequenced by the Illumina NovaSeq 6000 System platform according to the manufacturer’s instructions.

### 4.4. De novo Transcriptome Assembly and Gene Expression Comparison among Tissues

Paired-end short reads were assembled by Trinity [33], and the Tgicl was used to cluster transcripts into unigenes. All the clean reads were mapped onto the unigenes by using Bowtie2 [34], and the gene expression level was calculated with RSEM [35]. The gene expression levels were evaluated by FPKM (fragments per kilobase per million reads) values, with FPKM ≥ 0.5 as a cut-off value. To identify the DEGs (differentially expressed genes), DESeq2 was applied for the analysis.

The homologous genes and expression levels were analysed to investigate their involvement in RIN and IRN biosynthesis. Sequences of tryptophan decarboxylase (TDC), iridoid oxidase (IO), 7-deoxyloganetic acid glucosyltransferase (7-DLGT), 7-deoxyloganic acid hydroxylase (7-DLH), loganic acid O-methyltransferase (LAMT), secologanin synthase (SLS) and strictosidine synthase (STR) from Catharanthus roseus were used as probes to characterize the homologous genes in *U. rhynchophylla*. The expression levels of these functional genes and candidate genes (CYP450 and FMO) were used for co-expression analysis.

### 4.5. Functional Annotation for U. rhynchophylla

NCBI non-redundant proteins (Nr), NCBI nucleotide database (Nt), SWISS-PROT Protein Sequence Data Bank (SWISS-PROT), Kyoto Encyclopedia of Genes and Genomes (KEGG), Gene Ontology (GO) and PFAM database were used for gene annotation.

### 4.6. Phylogenetic Analysis

The protein sequences of characterized STRs were downloaded from the NCBI database. *Cr*STR was used as the probe to discover *Ur*STR by the local blast with the *U. rhynchophylla* transcriptome database. Then, all the sequences were aligned by the MUSCLE algorithm. The maximum likelihood method generated the phylogenetic trees with the JTT model and bootstrap values set to 1000 [36].

### 4.7. Plasmids Construction, Protein Purification

The total RNA from *U. rhynchophylla* was extracted with the MolPure^®^ Plant RNA Kit (Yeasen, Cat #19291ES50). The coding sequences of *Ur*STR were amplified by PCR from cDNA using the primers in Table S2 and inserted into the pET-30a expression vector. Escherichia coli strain DH5α was used as the cloning host for plasmid construction, and the correct constructs were introduced into *E. coli* BL21 (DE3) after validation by sequencing. The recombinant *Ur*STR proteins were purified following the general Ni-NTA purification procedure. The BL21 (DE3) strain containing *pET28a-UrSTR* was cultured for recombinant protein expression. We inoculated a 10 uL glycerol stock of the *Ur*STR strain into 5 mL LB medium and cultivated it at 37 °C overnight. Then, the seed cultures were transferred into 1 L of LB medium with kanamycin (50 mg/L) until the OD600 reached 0.6. For protein expression, we added 200 μM isopropyl-*β*-D-thiogalactoside (IPTG) to the cultures to induce protein production over 20 h at 16 °C. 

After collection by centrifugation at 6000× *g* under 4 °C, we suspended the cell pellets in 30 mL of lysis buffer (50 mM Tris-HCl, pH 8.0, 100 mM NaCl, 5 mM imidazole and 5% glycerol). The mixtures were lysed via sonication. After centrifugation at 15,000× *g* for 20 min, the supernatant was loaded onto a column with Ni-NTA resin. The lysis buffer containing increasing concentrations of imidazole (25 mM, 50 mM, 100 mM and 500 mM) was used to wash the column and elute the target protein. After SDS-PAGE analysis, the target fraction was concentrated and desalted by the PD-10 column, and a NanoDrop Spectrophotometer determined the protein concentration.

### 4.8. Enzymatic Assay and LCMS Analysis

To determine the function of *Ur*STR, the purified recombinant protein in Tris-HCl buffer (50 mM Tris-HCl, 50 mM NaCl, pH8.0) was used for the in vitro assay. In the total volume of 100 μL, 1 mM of secologanin and 2 mM of tryptamine/N-methyltryptamine/N,N-dimethyltryptamine were supplied with *Ur*STR. The mixtures were incubated at 30 °C for 2 h, then the reaction mixtures were incubated at 30 °C for one hour, and then they were quenched by adding an equal volume (100 μL) methanol with vertexing for 2 min. After centrifugation at 20,000× *g* for 15 min and filtration (0.22 um filter), the samples were subjected to LC-MS analysis.

The LC-MS method for analysis followed our previous study [32]. For analysis, an Agilent Eclipse plus C18 column (4.6 × 150 mm, 3.5 μm) was used on an Agilent 1260-6125+ LC-MS system. Mobile phases A (H_2_O containing 0.1% formic acid) and B (acetonitrile) were run in the gradient programme at 0.8 mL/min: 0–3 min, 5% B; 3–12 min, 5–30% B; 12–15 min, 30–95% B; 15–18 min, 95% B, 18–21 min, 95–5% B, 21–24 min, 5% B. A 10 μL sample was injected for analysis.

### 4.9. Structural and Docking Analyses for UrSTR

Homology modelling was performed with AlphaFold2 [37], using the *Rs*STR as the template (PDB ID: 4IYG). The results were inspected and rendered with PyMOL v2.5.2. Protein docking was done with AutoDock Vina using local search parameters and default docking parameters [38].

## 5. Conclusions

Global transcriptome profiling was conducted on four tissues of *U. rhynchophylla,* and a total of 35.44 Gb of clean data were generated. A total of 164,810 unigenes (>2000 bp) were assembled, and 141,747 unigenes (86%) were annotated through the public database. Our study successfully detected some Uncaria plant-specific indole alkaloids in *U. rhynchophylla*. Genes encoding the functional enzymes and transcriptional factors involved in the biosynthesis of spiro-indole alkaloids in *U. rhynchophylla* were identified in this study. In addition, we also characterized *Ur*STR and excluded the possibility for acceptance of N-methyltryptamine and N-dimethyltryptamine by biochemical approach and docking analysis. Moreover, some CYP450s and FMOs were proposed to be involved in the spiro-indole alkaloids, which still need to be characterized in the future.

In summary, the datasets provide a global transcriptome of *U. rhynchophylla* and pave the way to elucidate the biosynthetic pathway of Uncaria plants-specific indole alkaloids, especially rhynchophylline and isorhynchophylline. This study provides valuable genetic resources and catalytic elements for the biosynthetic pathway elucidation and synthetic biology of spiro-indole alkaloids.

## Figures and Tables

**Table 1:** Overview of the sequencing and assembly of transcriptome of *U. rhynchophylla*.

Items	RAW READS	Clean Reads	Clean Bases
Leaf	71,187,462	69,670,550	10.45 G
Stem Bark	56,685,480	55,496,154	8.32 G
Root	55,949,240	54,721,630	8.21 G
Bud	57,681,414	56,408,860	8.46 G
Total data	241,503,596	236,297,194	35.44 G
Unigenes ≥ 500 bp	311,204		
N50 (bp)	2887		

**Table 2:** Summary of unigenes’ annotations of *U. rhynchophylla*.

	Number of Unigenes	Percentage (%)
Annotated in NR	137,093	83.18
Annotated in NT	102,057	61.92
Annotated in KO	59,289	35.97
Annotated in SwissProt	109,519	66.45
Annotated in PFAM	102,231	62.02
Annotated in GO	102,231	62.02
Annotated in KOG	43,521	26.4
Annotated in Total	141,747	86

## Data Availability

The raw sequence data reported in this paper have been deposited in the Genome Sequence Archive in BIG Data Center, Beijing Institute of Genomics (BIG), Chinese Academy of Sciences. Other data supporting the results in this study are shown in the Supplementary Materials.

## Supplementary Materials

The following supporting information can be downloaded at: https://www.mdpi.com/article/10.3390/biom12121790/s1.
