# Supplementary material for: Profiles of Metabolic Genes in Uncaria rhynchophylla and Characterization of the Critical Enzyme Involved in the Biosynthesis of Bioactive Compounds-(iso)Rhynchophylline
Source: Biomolecules. 2022 Nov 30;12(12):1790. doi: 10.3390/biom12121790 (PMC9775700; doi:10.3390/biom12121790)

Figure S3. Content of representative indole alkaloids (rhyncholphylline and isorhyncholphylline) in different tissues of *U. rhynchophylla*.

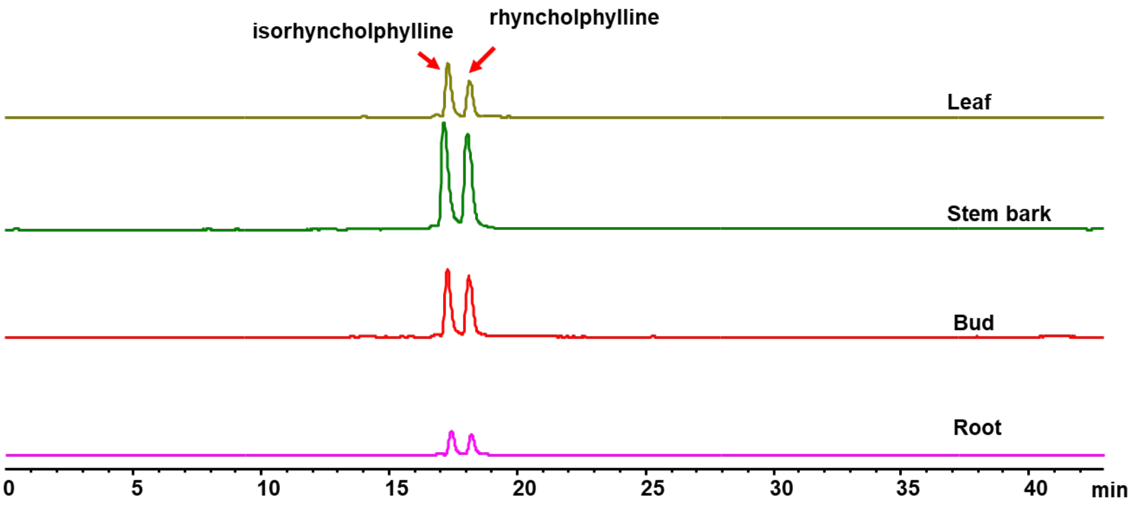

Supplement: Supplementary file 1 [file biomolecules-12-01790-s001.zip › biomolecules-1983171-supplementary- new/Supplementary Figure S3.pdf]
