# Supplementary material for: Profiles of Metabolic Genes in Uncaria rhynchophylla and Characterization of the Critical Enzyme Involved in the Biosynthesis of Bioactive Compounds-(iso)Rhynchophylline
Source: Biomolecules. 2022 Nov 30;12(12):1790. doi: 10.3390/biom12121790 (PMC9775700; doi:10.3390/biom12121790)
Supplement: Supplementary file 1 [file biomolecules-12-01790-s001.zip › biomolecules-1983171-supplementary- new/Supplementary Table S2.pdf]

Table S2. Gene sequences and primers list used in this study

>*Ur*STR

ATGCTCTCGGAAGTCAGGTTTAACATGCACAGTTCCATGGTTGCGTTAACCATTTTCCTTATCCTTTTCTGG
TCCCCTCTTTCCGTAGTTCTATCTTCTGCTGAATTTCTCCAGTTCATCAAGTCACCCTACGGCCCAAACGCC
TTCGCTTTTAACTCAGCTGGTGAACTTTTCGCTACCGTCGAAGATGGCAGAATTGTTAAGTATAATGGAAA
AGGACCGAGCAACGGGTTTTCGACCCACGCTGTTGCCTCTCCAGTCTGGAACAGAAAAGTTTGTGAGAAT
AATTCTAAACCTGAGCTAAAACCATTTTGTGGGAGGGCATATGACCTCGGATTTCACTATGAAACTCAGC
AATTGTATATTGTTGATTGTTATTATGGTCTTGGTGTGGTTGGACCTGAAGGTGGCCGTGTGACTCAGCTT
GCTAAGAGTGCAGATGGAAGGGACTTCAAGTGGCTTTATGCCTTGGCCGTGGACCAGCAAACTGGCTCT
TTGTACACCACAGATGTTAGCGCAAAATACGACGACAGAGGTGTTCAAGACATCCTGAGGATAAATGAT
TCAACAGGAAGATTAATCAAATATGATCCCTCAGCTAATGAAGCTAGCGTTTTATTGAAAGATCTGAATA
TACCAGGTGGTGTTGAACTTAGCCAAGATGGCTCATTTCTTCTTGTTGGTGAATACTTGAGCCACAGAATT
CTCAAGTATTGGTTAAAGGGCCCTAAGGCAAATACTTGGGAGGTATTTTTCAAAGTGAGGTTCCCAGGTA
ACATAAAAAGGAATAATGCTGGAGAATTTTGGATAGCCTCTAGTGACTATAATGGAATTACTGTTACGCC
TAGAGGTTTTAGGGTTGACGAATCTGCCAACGTCTTAGAAGTCGTGCCCATCCCTCCGCCATATAAAGGT
GAATTTTACGAACAAGTTCAAGAGCATAATGGTGCACTTTACGTCGGGTCTTTATTCCACGACTTTGTGG
GTATATTACATAATTACGAGGGGTCATCTGATCCCAAAGAAAATAATGCAGATGGGTTCAATGGATCTTT
GAATGGATTGGCTTCTTCTGTC

Primer Name	Sequence
*Ur*STR-BamHI-F	CGCGGATCCATGCTCTCGGAAGTCAGGTTTAACATG
*Ur*STR-SalI-R	ACGCGTCGACCTAGACAGAAGAAGCCAATCCATTC
